# A Case Report of Steroid-Resistant Cryptogenic Organizing Pneumonia Managed with Intravenous Immunoglobulins

**DOI:** 10.1155/2021/9343491

**Published:** 2021-11-15

**Authors:** Christian Akem Dimala, Urvi Patel, Benjamin Lloyd, Anthony Donato, William B. Kimmel, Robert Hallowell, Caitlyn Moss

**Affiliations:** ^1^Department of Medicine, Reading Hospital-Tower Health, West Reading, PA, USA; ^2^Department of Pathology, Reading Hospital-Tower Health, West Reading, PA, USA; ^3^Department of Pulmonary & Critical Care Medicine, Massachusetts General Hospital, Boston, MA, USA

## Abstract

Fewer than ten reported cases of cryptogenic organizing pneumonia (COP) have been managed with intravenous immunoglobulins (IVIg). We report a case of a 72-year-old man who presented with a worsening cough and diffuse opacities on chest radiograph. Following no improvement with antibiotics and negative complementary investigations for infectious, malignant, and autoimmune etiologies, COP was confirmed on lung biopsy. Due to continued clinical deterioration despite high-dose steroids and new severe steroid-induced hallucinations, the patient was placed on intravenous immunoglobulins (IVIg) and mycophenolate mofetil and made a satisfactory recovery. IVIg should be considered as an important steroid-sparing alternative in patients with COP.

## 1. Introduction

Cryptogenic organizing pneumonia (COP) is a rare respiratory condition characterized by the filling of alveolar airspaces and alveolar ducts with fibrin and granulation tissue, and for which there is no identifiable cause [1, 2]. COP often presents with respiratory symptoms including cough, fever, shortness of breath, and fatigue; however, its clinical presentation could span through a wide spectrum [1–3]. Lung examination is usually remarkable for focal or widespread crackles. Radiologic changes often observed are unilateral or bilateral lung opacities on chest radiographs and ground glass shadowing on computed tomography (CT) scans [4, 5]. Typical histopathologic features are patchy cellular airspace fibrosis involving alveoli and alveolar ducts, foamy alveolar macrophages, and fibroblasts embedded in a myxoid matrix [4]. The mainstay of management of COP involves corticosteroids. Improvement in symptoms and oxygen requirements is seen in more than half of the cases [3] and clinical improvement over several months [1]. There have been reports of corticosteroid-resistant and refractory cases of COP requiring alternate management options including macrolides and cytotoxic agents [6, 7]. Steroid pulse therapy with high doses of methylprednisolone has also been reported [8–10]. However, intolerance to high-dose steroids in some patients warrants alternative treatment options. We report a case of corticosteroid-resistant COP in a patient who developed steroid psychosis, requiring treatment with intravenous immunoglobulins (IVIg).

## 2. Case Presentation

**Figure 1:**
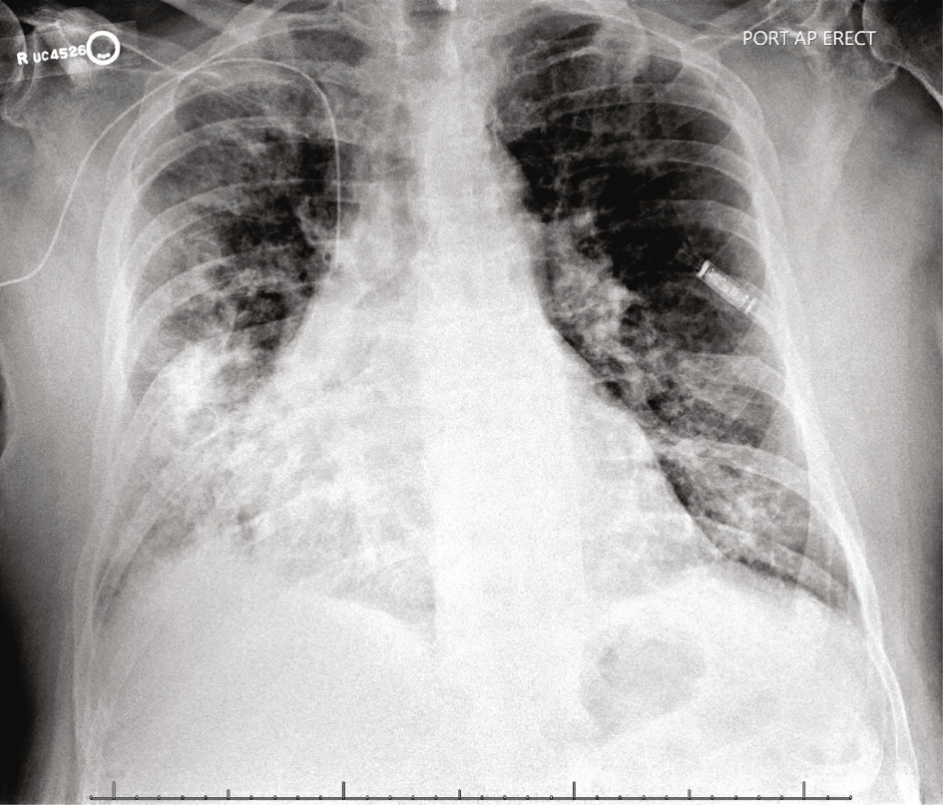
Anteroposterior erect chest radiograph at presentation. Frontal view of chest radiograph showing bilateral patchy infiltrates most pronounced in the right lower lobe concerning for multifocal pneumonia. Right PICC line and cardiac recorder device noted in the left anterior chest wall.

**Figure 2:**
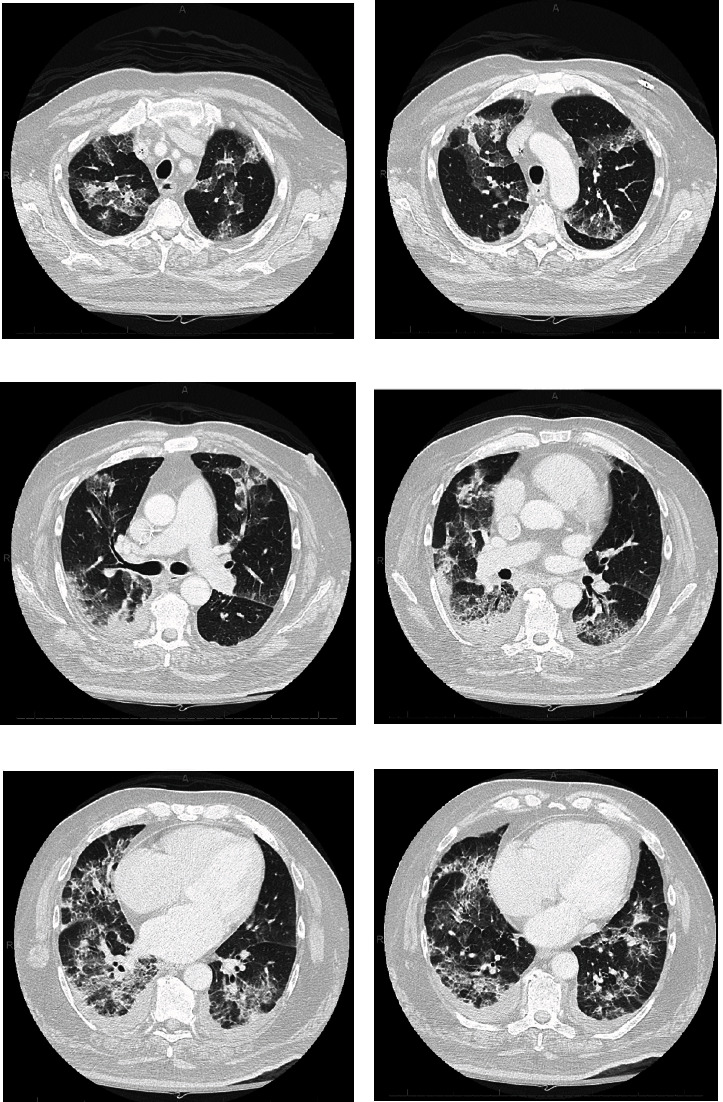
CT scan of the chest with intravenous contrast at day four of admission. CT chest images (a)–(f) showing patchy opacities of both lungs involving all lobes bilaterally (right greater than left) with some consolidation changes and mild bilateral pleural effusions.

**Figure 3:**
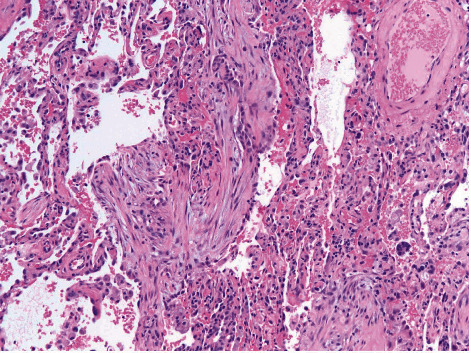
Surgical biopsy obtained from right lung. Pathology with hematoxylin-eosin stain at 200× magnification shows organizing pneumonia in a background of diffuse alveolar damage.

**Figure 4:**
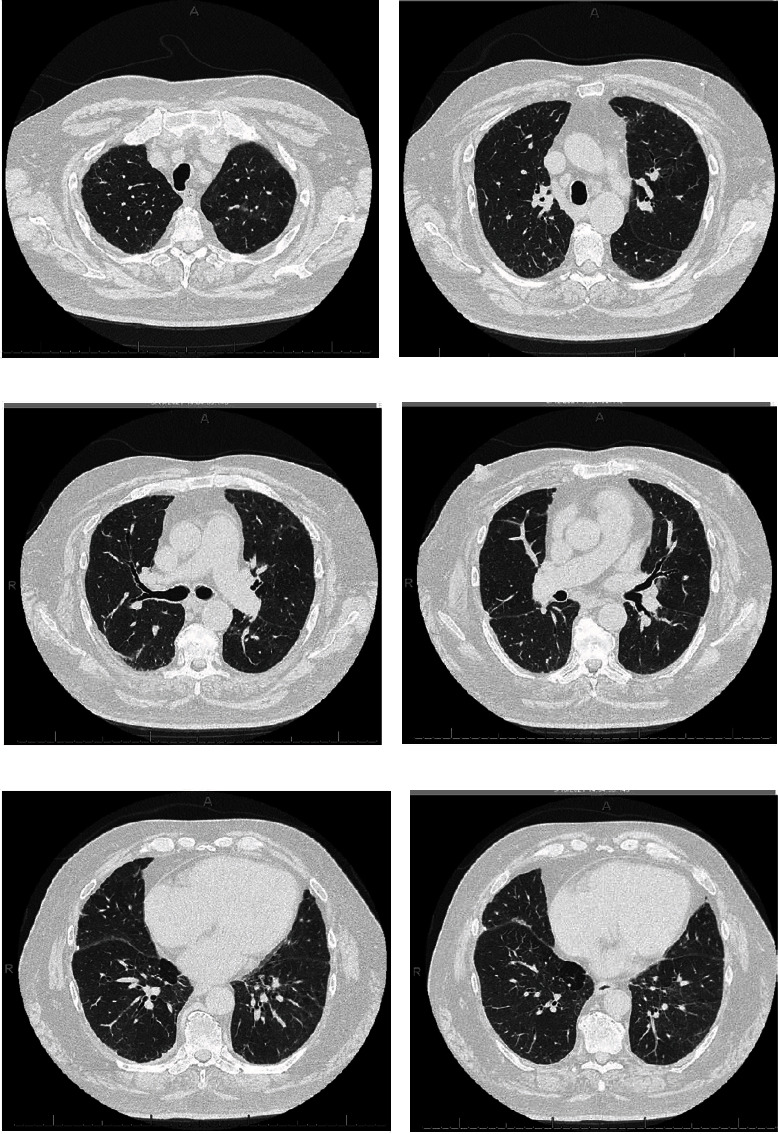
CT scan of the chest with intravenous contrast at 6-month follow-up visit. CT chest images (a)–(f) showing significant interval improvement of ground glass opacities and interstitial infiltrates in the lungs with just a few markings remaining in the lung bases, suggesting interval resolution of the inflammatory process.

**Figure 5:**
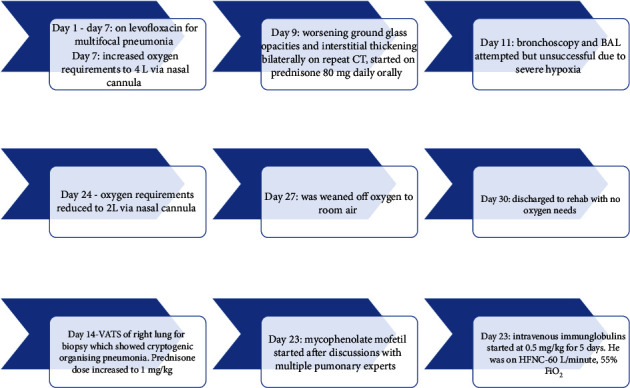
Flow chart summarizing hospital course of patient.

A 72-year-old man with atrial fibrillation on apixaban and a left total knee replacement four weeks prior, complicated by hemarthrosis and joint infection requiring arthrocentesis and currently receiving a 6-week course of intravenous daptomycin presented to the hospital from a rehabilitation facility due to worsening cough for over one week. Chest radiograph showed patchy infiltrates, most pronounced in right lower lobe suggestive of multifocal pneumonia (Figure 1). He complained of intermittent productive cough with one episode of hemoptysis, as well as dyspnea and chills. On presentation, the patient appeared comfortable. However, he was febrile to 100.4 degrees Fahrenheit and tachypneic. On examination, heart rhythm was irregularly irregular, there was no jugular venous distension, and the patient had bilateral rales from lung bases up to mid-lung zones. No lower extremity edema was noted. His left knee surgical incision site was intact and clean, with minimal sanguineous drainage and minor surrounding erythema. Laboratory studies were notable for white blood cell count of 10,900 cells/*μ*L with 4.7% eosinophils and 69.7% neutrophils, hemoglobin of 11.0 g/dL, sedimentation rate of 22 mm/hour (normal < 15 mm/hr), and c-reactive protein of 7.23 mg/dL (normal < 1.00 mg/dL). Chemistries were significant for a sodium of 129 mmol/L. The patient was admitted for presumed sepsis secondary to multifocal pneumonia and treated with cefepime and metronidazole for pneumonia, and he continued with daptomycin for septic arthritis. Cefepime and metronidazole were subsequently switched to levofloxacin for seven days to treat community-acquired pneumonia. Despite antimicrobial therapy, the patient gradually deteriorated with worsening respiratory failure. On day four of his hospital stay, a computed tomography (CT) scan of the chest demonstrated ground glass opacities involving all lobes bilaterally with consolidation at bilateral lung bases (Figure 2). Due to concern for daptomycin-induced eosinophilic pneumonitis, the patient was switched to intravenous vancomycin for septic arthritis antimicrobial coverage. Seven days after admission, the patient had increasing oxygen requirements, requiring four liters of oxygen. Transthoracic echocardiogram from the current admission demonstrated an ejection fraction of 55% with no regional wall motion abnormalities. B-type natriuretic peptide was initially raised at 486 pg/mL, but subsequently declined to 179 pg/mL on day seven of admission and 111 pg/mL on day 15 of admission. Multiple trials of diuresis were attempted due to suspicions of volume overload with a net negative fluid balance of 12.4 liters over 17 days. Despite attempted diuresis, there was no change in oxygen requirements, and his repeat chest X-ray still demonstrated bilateral patchy infiltrates. A repeat CT of the chest demonstrated worsening ground glass opacities and small bilateral pleural effusions. All investigations including two coronavirus-19 nucleic acid amplification (NAA) tests and respiratory pathogen panel were negative. Blood cultures were negative. Urine *Legionella* and *Streptococcal pneumoniae* antigens, as well as serum mycoplasma IgM antibody were negative. HIV antibody/antigen testing was negative. Procalcitonin was 0.07 ng/mL (normal < 0.5 ng/mL). Patient's inflammatory markers were, however, increasing with C-reactive protein at 20.8 mg/L, erythrocyte sedimentation rate of 38 mm/hr, and white blood cell count to 14,700/*μ*L with 4.2% eosinophils and 76.9% neutrophils on day seven of admission. Rheumatic and autoimmune laboratories were negative including anti neutrophilic cytoplasmic antibody, rheumatoid factor, myeloperoxidase antibody IgG, serine proteinase antibody, dsDNA antibody, and autoantibodies to SSA and SSB. Antinuclear antibody was positive at 1 : 80, speckled. The workup for hypersensitivity pneumonitis was negative for antibodies to *Aspergillus*, *Thermoactinomyces vulgaris*, and *Micropolyspora faeni*. The patient was transferred to an intermediate care unit due to worsening respiratory failure. Pulmonology was consulted and recommended initiation of prednisone 1 mg/kg per day for potential cryptogenic organizing pneumonia. Despite steroid therapy, the patient's respiratory status continued to decline, requiring up to 10 liters of oxygen via oxi-mask to maintain oxygen saturations above 92%. A limited bronchoscopy with bronchoalveolar lavage was completed on day 11 but biopsies could not be obtained due to respiratory distress. Fluid studies from bronchoalveolar lavage showed white blood cell count of 355 c/mm^3^ with 36% segmented cells, 8% lymphocytes, 16% monocytes, and 3% eosinophils. Bronchial culture was suggestive of oral flora and negative for acid fast bacilli. Bronchial fungal culture was positive for *Candida albicans* but was thought to be a contaminant. Cytology from the bronchial washing was negative for malignancy. On day 14, the patient underwent video-assisted thoracoscopic surgery for lung biopsy that was complicated by pneumothorax requiring chest tube placement. His respiratory status worsened with arterial blood gas demonstrating hypercapnia despite continuing 1 mg/kg of steroids (80 mg prednisone orally). Oxygen requirements escalated to 60 liters O_2_ via high flow nasal canula at 55% FiO_2_. His hospital course was further complicated by steroid-associated psychosis and delirium requiring physical and chemical restraints, and the patient was hence deemed unsafe for BiPAP. On hospital day 18, lung biopsy result was consistent with cryptogenic organizing pneumonia (Figure 3). Given worsening respiratory status, medications to induce remission were discussed. Given severe delirium and psychosis from steroids, intravenous immunoglobulin (IVIg) 0.4 g/kg/dose was initiated on day 23 and continued for five days as an alternative to pulse steroids based on multiple case reports. Mycophenolate mofetil 500 mg twice daily was also initiated to wean down prednisone, which was decreased to 0.4 mg/kg after a one-time dose of methylprednisolone 125 mg on day 23. Trimethoprim-sulfamethoxazole was initiated for *Pneumocystis jirovecii* pneumonia prophylaxis. The patient made a significant improvement after initiation of IVIg with complete resolution of acute hypoxic respiratory failure and ended his oxygen dependence by day 4 of IVIg (hospital day 27). The patient was discharged on an eight-week prednisone taper, vitamin D and calcium supplementation, mycophenolate mofetil, and trimethoprim sulfamethoxazole. A follow-up chest CT scan of the patient at 6 months showed significant interval improvement of the ground glass opacities and interstitial infiltrates in the lungs with just a few markings remaining in the lung bases (Figure 4), suggesting interval resolution of the inflammatory process. A summary of the patient's hospital course is presented on Figure 5.

## 3. Discussion

Cryptogenic organizing pneumonia (COP) was first described in 1983 by Davison et al. in eight patients who presented with respiratory symptoms, bilateral radiographic changes, elevated inflammatory markers, and intra-alveolar organization but no identifiable etiology [11]. The disease is believed to be caused by injury to the alveolar epithelium with subsequent deposition of fibrin [1, 2]. COP classically presents with nonspecific symptoms, including a subacute cough lasting weeks to months associated with fever, shortness of breath, malaise, fatigue, and sparse crackles on auscultation with multifocal alveolar infiltrates on chest imaging [1, 5]. Typical radiological findings on chest CT scan include multifocal airspace opacities or peripheral consolidations with by ground glass opacities [12]. Atypical radiological patterns include infiltrative opacities, multiple nodular opacities, crazy-paving patterns, and linear and band-like opacities among others [12]. Symptoms with similar clinical and histologic features include infections, autoimmune diseases, connective tissue diseases, and drug injury among others. It is important to exclude all these causes to be able to arrive at the diagnosis of COP. Untreated typical COP has a good prognosis with spontaneous recovery in up to half of the cases [3]. Treated COP has an even better long-term outcome in most cases with significant improvement in symptoms within days [1, 3]. Cases with interstitial fibrosis as opposed to those with airspace opacities on chest imaging, however, have persistence of the disease even with treatment [1, 3]. Current treatment guidelines for COP are based on the severity of the disease at presentation and include observation for mild disease with minimal symptoms or oral glucocorticoids for patients with worsening symptoms, moderately decreased pulmonary functions tests, and diffuse radiographic changes. After initial loading doses of 500-1000 mg of intravenous methylprednisolone for three days, the recommended maintenance doses of steroids are 0.75-1.5 mg/kg prednisone for up to three months with gradual weaning over several months and potentially up to a year, if there is satisfactory clinical improvement [2, 3]. The side effects of steroids should always be kept in mind when used on a long-term basis, and these patients should be followed-up regularly with repeat radiographic imaging to ensure nonprogression or relapse of the disease that will require steroid dose adjustment or an alternative treatment.

Although steroids are the main treatment modality for moderate to severe COP^2^, complete response to steroids has been observed in only 60% of cases, with nonresponse noted in up to 14% of cases [3]. Likewise, relapses are not infrequent, especially when the steroid dose is rapidly tapered. Unfortunately, the side-effect profile of steroids makes them unsuitable in some individuals such as those with steroid-induced psychosis as was the case in this patient. In these cases, alternative treatment options should be sought. Antibiotics with anti-inflammatory properties such as macrolides have been increasingly used for the treatment of patients with COP usually administered over a period of several months or up to a year [7, 13]. Other treatment modalities which have been used previously include adjunctive immunosuppression therapy with cyclophosphamide [14], cyclosporine [15], azathioprine [16], and mycophenolate mofetil [17]. In our case, macrolide therapy was not attempted.

Another important etiology considered in this patient was daptomycin-induced lung injury as he had been on daptomycin. A case of organizing pneumonia with pulmonary eosinophilic infiltrates following prolonged use of daptomycin in a very similar context has previously been reported [18]. In the former case report, clinical improvement was achieved within a couple of weeks, following discontinuation of daptomycin. However, there was rather a persistent clinical deterioration in our patient after discontinuation of daptomycin, and there was no eosinophilia on differential blood count or evidence of eosinophilic inflammation on bronchoalveolar lavage and lung biopsy. He was started on high-dose prednisone when COP became a likely diagnosis but showed no improvement on steroids, and a decision was made to start the patient on intravenous immunoglobulins to which he responded within 24 hours and experienced complete recovery and discharge over the following days. Although mycophenolate mofetil was also started at the same time as IVIg, we believe IVIg to be responsible for his improvement given that the effects of antimetabolites such as mycophenolate mofetil are generally not seen for several weeks. To date, we found only seven published cases that used intravenous immunoglobulins (IVIg) for the treatment of organizing pneumonia, often in the context of underlying selective immunodeficiency or hypogammaglobulinemia and/or nonresponse to steroid [19–25]. These cases suggest that a normal and functional humoral immune response is not an absolute requirement for the development of COP and also raise the possibility of an association of COP to hypogammaglobulinemia in the setting of repeated infections. However, further research is needed in this area. Our patient did not have features suggestive of underlying immunodeficiency such as recurrent or opportunistic infections. This patient was discharged with scheduled outpatient follow-up visits given the high relapse rates of COP especially in cases presenting with multifocal opacities on radiologic test [26].

## 4. Conclusion

1. Cryptogenic organizing pneumonia remains a rare respiratory entity, typically managed with corticosteroids
2. Intravenous immunoglobulins should be considered as an important steroid-sparing alternative treatment even in patients without underlying immunodeficiencies or hypogammaglobulinemia

## Data Availability

Data sharing is not applicable to this article as no new data were created or analyzed in this study.

## Abbreviations

COP: Cryptogenic organizing pneumonia
CT: Computed tomography
IVIg: Intravenous immunoglobulins
NAA: Nucleic acid amplification.
